# Association between anterior bone loss and anterior heterotopic ossification in hybrid surgery

**DOI:** 10.1186/s12891-020-03664-w

**Published:** 2020-10-08

**Authors:** Junbo He, Hao Liu, Tingkui Wu, Chen Ding, Kangkang Huang, Ying Hong, Beiyu Wang

**Affiliations:** Department of Orthopedic Surgery, West China Hospital, Sichuan University, No. 37 Guo Xue Rd, Chengdu, 610041 China

**Keywords:** Anterior bone loss, Anterior heterotopic ossification, Hybrid surgery

## Abstract

**Background:**

Hybrid surgery (HS) has become an alternative procedure for the treatment of multilevel cervical degenerative disc disease with satisfactory outcomes. However, some adverse outcomes have recently emerged, such as heterotopic ossification (HO) and anterior bone loss (ABL). Furthermore, HO was found mostly located in the posterior and lateral of the cervical intervertebral disc space. The mechanism of anterior heterotopic ossification (AHO) formation may be different, and its relationship with ABL was uncertain.

**Methods:**

Radiographical and clinical outcomes of ninety-seven patients who had undergone contiguous two-level HS between December 2010 and December 2017 and with a minimum of 2-year follow-up were analyzed. Postoperative radiographs were evaluated and compared to the initial postoperative films to determine the incidence of ABL and AHO.

**Results:**

The overall incidence rate of ABL was 44.3% (43/97). It was identified in 70.6% of AHO cases (33.3% mild, 41.7% moderate, 25.0% severe) and 38.8% of non-AHO cases (38.7% mild, 45.2% moderate, 16.1% severe). A significant association between ABL and AHO occurrence was found (*P* = 0.016). There was no significant difference in prosthesis–endplate depth ratio or disc space angle change between the AHO group and the non-AHO group (*P* > 0.05). Compared with data preoperatively, clinical outcome scores significantly improved after surgery in both the AHO and non-AHO groups, with no significant differences between the two groups (*P >* 0.05).

**Conclusion:**

ABL was common in HS, and it related to AHO. The formation of AHO could be an integral part of postoperative bone remodeling, as well as ABL.

## Introduction

Cervical degenerative disc disease (CDDD) is a common, age-related, and progressive disorder that can present with mechanical neck pain, radiculopathy, myelopathy, or a combination of these symptoms. Surgery is generally indicated to treat these patients when conservative treatments fail and has yielded satisfactory clinical outcomes via several approaches [1]. Compared with traditional anterior cervical discectomy and fusion (ACDF), cervical disc arthroplasty (CDA) has been demonstrated to preserve the range of motion (ROM) at the operated level and thereby decrease the incidence of adjacent segment pathology [2–4]. However, the surgical indications of CDA are relatively narrow and not acceptable for all diseased levels. Meanwhile, multilevel CDA might add difficulty to the technique, increase the possibility of disc prostheses complications with increasing implant levels, and increase medical costs [5–7]. In this regard, hybrid surgery (HS), consisting of CDA at the mobile level and ACDF at the spondylotic level, has been introduced as an alternative procedure for the treatment of multilevel CDDD with satisfactory outcomes [7–9].

However, few studies have documented some adverse outcomes, including device displacement, expulsion, loosening or fracture, heterotopic ossification (HO), and anterior bone loss (ABL), were identified in CDA and HS [1, 6, 9–15]. The mechanism of HO and ABL formation remains unknown, but studies have suggested that the occurrence of HO and ABL were both related to changes in biomechanical environment [16, 17]. Furthermore, in vivo and in vitro studies have confirmed that fusion segments in HS significantly affected the biomechanical environment of its adjacent CDAs [5, 13], which may explain the higher incidence of HO and ABL after HS [13, 14]. Moreover, HO was found mostly located in the posterior and lateral of the cervical intervertebral disc space [15]. The mechanism of anterior heterotopic ossification (AHO) formation may be different, and its relationship with ABL was uncertain. The purpose of our study was to investigate the correlation between ABL and AHO after HS.

## Methods

### Patients

**Fig. 1:**
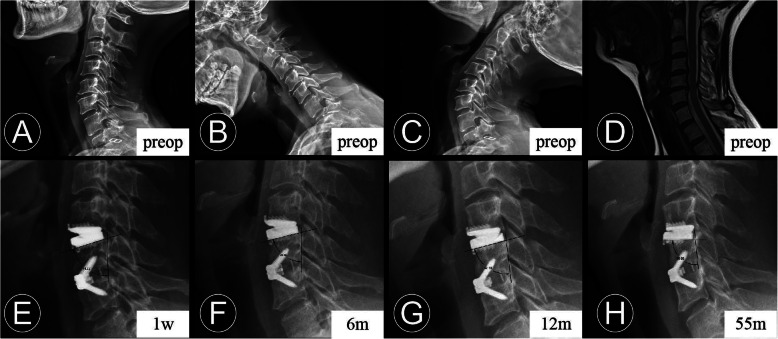
A 51-year-old woman who underwent contiguous 2-level hybrid surgery at C4-C6. **b** and **c** Preoperative dynamic radiographs show that a loss of intervertebral disc height and decrease of segmental mobility occurred at C5–6. Thus, fusion was performed at C5–6 and cervical disc arthroplasty was performed at C4–5. **e** and **f** The immediate and 6-month postoperative lateral radiographs show the peri-prosthesis bone loss was found at the replacement level. **e**, **f**, **g**, and **h** The measurement method for the degree between the prosthesis position and posterior vertebral line. The postoperative lateral radiographs obtained at 1 week, 6 months, 12 months, and 55 months after surgery show the progression of implant subsidence

We retrospectively reviewed 97 patients (34 men and 63 women) who underwent two-level HS between December 2010 to December 2017 and had a minimum of 24 months follow-up. All patients provided written informed consent, and the study protocol was approved by the Ethics Committee of West China Hospital of Sichuan University. Patients enrolled had been diagnosed with contiguous 2-level cervical degenerative disc disease with symptomatic radiculopathy and/or myelopathy. In the same line, the patients had not responded to conservative treatment for more than 6 weeks at 2 contiguous levels from C-3 to C-7 based on symptoms, signs, preoperative static and dynamic radiographs, computed tomography scans, and magnetic resonance imaging findings. The exclusion criteria consisted of any prior spine surgery, ossification of the posterior longitudinal ligament, severe facet arthritis, cervical stenosis, fracture, infection, tumor, and osteoporosis. CDA was performed at the segment without cervical instability (sagittal plane translation > 3.5 mm and/or sagittal plane angulation > 20°), with a disc height loss < 50%, without absence of motion < 2° and without facet joint degeneration. If radiographic signs of instability, bridging osteophytes and facet degeneration were observed, ACDF was chosen (Fig. 1).

### Surgical techniques

All operations were performed by the same senior spine surgeon. The patient was placed with the neck in a neutral position after general anesthesia. A standard right-side incision was performed along the skin crease to access the anterior cervical spine. Discectomy and decompression were performed using an anterior approach. The more severe degenerative segment should be decompressed primarily. CDA procedures were performed using a proper size Prestige-LP (Medtronic Sofamor Danek, Memphis, TN). For ACDF procedures, the Zero-P (Synthes, Oberdorf, Switzerland) implants packed with β-tricalcium phosphate or local excised bone were inserted into the well-prepared intervertebral space. All prostheses were placed under fluoroscopic guidance. After surgery, all patients were instructed to perform neck function training within the first 3 weeks and immobilized with a collar for 4 to 12 weeks.

### Data collection

All clinical and radiographical outcomes were routinely collected preoperatively and at routine postoperative intervals of 1 week, 3 months, 6 months, 12 months, and at the final follow-up. Arm and neck pain of the patients were assessed by visual analogue scale (VAS) scores. The neck disability index (NDI) scores were used to evaluate the function of the neck. The Japanese orthopaedic association (JOA) scores were used to assess the neurological status of patients with myelopathy. Radiographical evaluations were conducted via lateral radiographs under flexion and extension and in a neutral position. The angle of cervical lordosis (CL), ROM of the whole cervical spine, disc angle, ROM of the arthroplasty segment, FSU height, and endplate length were measured as described in previous studies [14, 18].

The change in arthroplasty disc angle was defined as the difference between the preoperative and immediate postoperative arthroplasty disc angle values (immediate postoperative value minus the preoperative value) [19]. The prosthesis-endplate depth ratio was calculated on the lateral radiograph. It was calculated by dividing the length of the prosthesis by the immediate postoperative length of the endplate [20]. The change in arthroplasty disc angle was defined as the difference between the preoperative and immediate post-operative arthroplasty disc angle values (immediate post-operative value minus the preoperative value) [19]. The prosthesis-endplate depth ratio was calculated on the lateral radiograph. It was calculated by dividing the length of the prosthesis by the immediate post-operative length of the endplate [20].

**Fig. 2:**
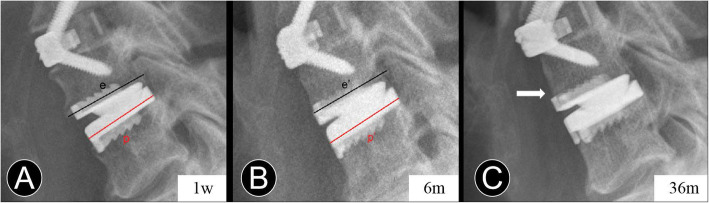
Measurement of anterior bone loss. **a** and **b** Immediate and 6-month postoperative lateral radiographs, p and p’ are the length of the prostheses. e and e’ are the length of the endplates. EL% = 100% × (e / p) / (e’ / p’). **c** At the last follow-up, the lateral radiograph shows peri-prosthesis bone loss at the anterior vertebral body margin (white arrow)

**Fig. 3:**
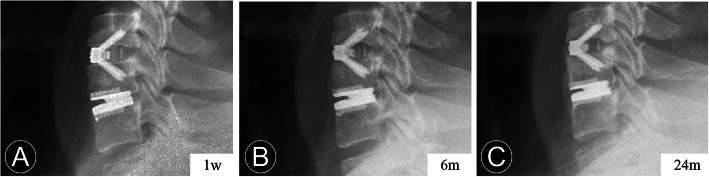
Serial postoperative radiographs of a 40-year-old man who underwent contiguous 2-level hybrid surgery at C5-C7. **a** and **b** The immediate and 6-month postoperative lateral radiographs show the peri-prosthesis bone loss and anterior heterotopic ossification. **c** At the last follow-up, the lateral radiograph shows the anterior heterotopic ossification developed

**Fig. 4:**
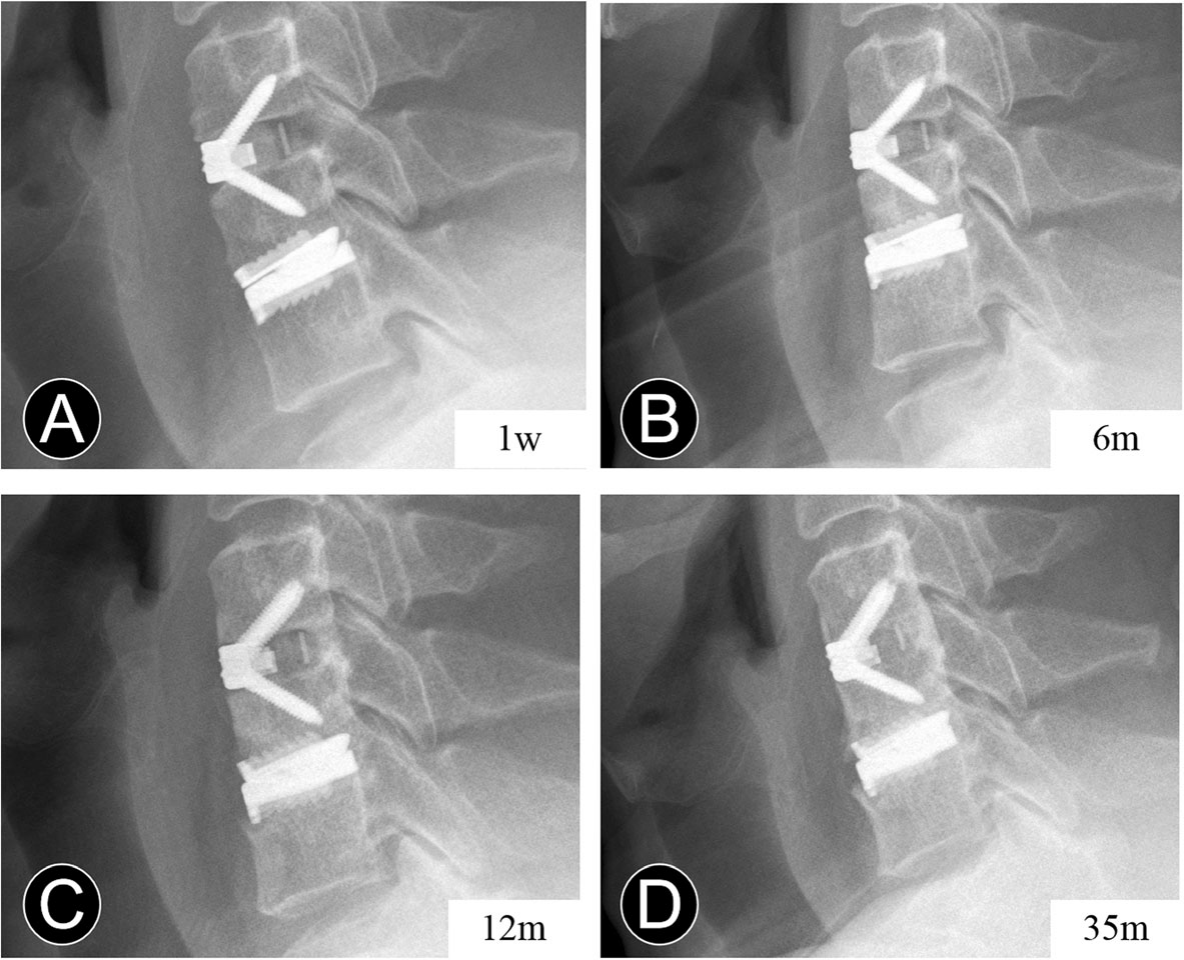
Serial postoperative radiographs of a 47-year-old man who underwent contiguous 2-level hybrid surgery at C5-C7. **a** and **b** The lateral radiographs show the anterior bone loss occurred at 6 months after surgery. **c** and **d** The 12-month and 35-month postoperative lateral radiographs show the anterior heterotopic ossification occurred after the non-progressive bone loss

ABL was identified as a combined standard of the changes in endplate length and implant subsidence at follow-up compared with immediately postoperatively at the arthroplasty level [14, 21]. It was determined on the lateral radiograph and divided into four grades based on Kieser’s classification and grading system (Table 1; Fig. 2). Endplate collapse or implant subsidence was defined as more than 5° change between the prosthesis position and posterior vertebral line when compared with that of the immediate postoperative radiograph (Fig. 1). AHO is defined as the abnormal presence of the bone in front of the CDA prosthesis (Figs. 3 and 4).

**Table 1:** Classification and grading system for ABL

Grade	Definition
None	No any peri-prosthetic bone loss
Mild	EL%^a^ > 95%
Moderate	EL% = 90–94%
Severe	EL% < 90% or implant subsidence

*ABL* anterior bone loss

^a^EL% = The endplate length at follow-ups / The immediate postoperative endplate length

All imaging examinations were independently assessed by 2 spine surgeons and 1 radiologist. When there was a difference in the imaging diagnosis between the two spine surgeons, the radiologist would use the Picture Archiving and Communication System imaging system to make the final decision.

### Statistical analysis

Standard statistical analysis was used for this study. SPSS software version 24.0 (SPSS, Chicago, IL, USA) was used for statistical analyses. A two-sided value of 0.05 was considered statistically significant. The results are presented as the mean  ±  standard deviation (SD) when the data satisfied the criteria for normality. Otherwise, the results are presented as the median ± interquartile range (IQR). A paired t-test was used to compare between preoperative and postoperative parameters. The independent t test or the nonparametric Mann–Whitney U test was used to compare quantitative data between the two groups, depending on whether the data were normally distributed. The chi-squared test or Fisher’s exact text was used to compare qualitative data between two groups. The inter-observer reliability of qualitative data was evaluated using Weighted kappa or Cohen kappa statistics.

## Results

### Patient populations

All 97 patients completed the follow-up, with an average follow-up duration of 37.6 months (range, 24–82 months). The mean operative time was 140.2 min, and the mean blood loss was 66.7 mL. The involved arthroplasty levels were C2/3 in 2 cases, C3/4 in 44 cases, C4/5 in 24 cases, and C6/7 in 27 cases. At the last follow-up, the incidence of HO and AHO were 66.0% (64/97) and 17.5% (17/97), respectively. With respect to perioperative parameters, there were no significant differences in the age, sex, BMI, BMD, pre- and post-ALP, operative level, blood loss, operation time, or follow-up time between AHO group and non-AHO group (*P* > 0.05) (Table 2).

**Table 2:** Demographic and perioperative characteristics

Variables	AHO Group	Non-AHO Group	*P* Value
No. of patients (n)	17	80	
Age (years)	48.7 ± 7.2	48.5 ± 6.8	0.927
Gender (M/F)	5 / 12	29 / 51	0.781
BMI (kg/m^2^)	24.9 ± 2.8	24.2 ± 3.0	0.398
BMD T value (spine)	0.2 ± 1.3	0.5 ± 1.2	0.352
Serological indicator			
Pre-ALP	71.6 ± 26.0	66.4 ± 18.5	0.331
Post-ALP	62.1 ± 21.3	59.2 ± 15.8	0.521
Arthroplasty segment			0.736
C3/4	0	2	
C4/5	8	36	
C5/6	3	21	
C6/7	6	21	
Fusion location			0.591
Up	8	31	
Down	9	49	
Operation time (minutes)	150.0 ± 26.8	134.6 ± 24.4	0.324
Blood loss (ml)	69.4 ± 48.1	66.1 ± 48.0	0.799
Follow-up time (months)	37.0 ± 17.3	37.7 ± 13.7	0.851

*AHO* anterior heterotopic ossification, *BMI* body mass index, *BMD* bone mineral density, *Pre-ALP* preoperative alkaline phosphatase, *Post-ALP* Immediate postoperative alkaline phosphatase

* *P* < 0.05

### Clinical outcomes

Compared with preoperative values, mean JOA, NDI, and VAS scores significantly improved after surgery in both groups, and remained highly improved throughout the follow-up period (*P* < 0.05). Moreover, there were no significant differences between the two groups at each time of evaluation (*P >* 0.05). The main clinical outcomes are presented in Table 3.

**Table 3:** Clinical outcomes

Variables	AHO Group(*n* = 17)	Non-AHO Group (*n* = 80)	*P* Value
JOA scores			
Preoperative	10.9 ± 1.8	11.3 ± 1.6	0.440
Postoperative 3 month	15.3 ± 0.8*	15.3 ± 0.9*	0.977
Last follow-up	16.1 ± 0.6*	16.2 ± 0.8*	0.811
NDI scores			
Preoperative	31.1 ± 3.2	30.0 ± 3.9	0.294
Postoperative 3 month	13.3 ± 2.7*	12.8 ± 3.4*	0.562
Last follow-up	8.8 ± 3.0*	7.9 ± 3.6*	0.325
VAS scores			
Preoperative	6.9 ± 1.1	6.5 ± 1.3	0.193
Postoperative 3 month	2.2 ± 0.8*	2.2 ± 0.9*	0.879
Last follow-up	1.4 ± 1.0*	1.2 ± 1.0*	0.528

*AHO* anterior heterotopic ossification, *JOA* Japanese Orthopedic Association, *NDI* Neck Disability Index, *VAS* Visual analog scale

* *P* < 0.05, compared with pre-operation

### Radiographical outcomes

#### Cervical lordosis and C2-C7 ROM

A summary of the radiographical outcomes and changes during the follow-up period are showed in Table 4. At the final follow-up, the CL in the AHO group and non-AHO group was increased to 8.7° ± 9.1° and 11.5° ± 12.3°, respectively. No significant difference was found between the two groups (*P* = 0.629). Due to the fusion segment, the ROM for C2-C7 in the AHO group was significantly decreased from 51.8° ± 15.7° preoperatively to 47.7° ± 8.9° at the last follow-up. In the non-AHO group, the ROM for C2-C7 was 46.9° ± 14.4° preoperatively and 42.7° ± 10.1° at the last follow-up with a significant decreasing (*P* < 0.05). However, there was no statistically significant difference between two groups (*P* = 0.148).

**Table 4:** Radiographical outcomes

Variables	AHO Group (*n* = 17)	Non-AHO Group (*n* = 80)	*P* Value
Cervical lordosis (°)			
Preoperative	5.3 ± 11.2	6.8 ± 10.8	0.611
Postoperative 3 month	7.9 ± 9.4*	10.2 ± 9.7*	0.384
Last follow-up	8.7 ± 9.1*	11.5 ± 12.3*	0.629
ROM C2-C7 (°)			
Preoperative	51.8 ± 15.7	46.9 ± 14.4	0.209
Postoperative 3 month	36.6 ± 9.8*	34.9 ± 10.8*	0.559
Last follow-up	47.7 ± 8.9*	42.7 ± 10.1*	0.148
Arthroplasty Disc Angle (°)			
Preoperative	1.7 ± 3.6	2.7 ± 3.5	0.313
Postoperative 3 month	2.2 ± 3.7	2.7 ± 4.5	0.681
Last follow-up	2.1 ± 5.2	3.4 ± 5.5	0.651
Arthroplasty Disc ROM (°)			
Preoperative	8.7 ± 3.3	9.4 ± 4.1	0.459
Postoperative 3 month	6.8 ± 4.1*	7.8 ± 4.8*	0.461
Last follow-up	8.1 ± 5.0	9.0 ± 5.1	0.537
Arthroplasty FSU (mm)			
Postoperative 1 week AH	27.6 ± 2.5	28.1 ± 2.9	0.496
Postoperative 1 week PH	27.5 ± 2.2	28.3 ± 2.7	0.251
Postoperative 3 month AH	27.3 ± 2.8	27.1 ± 4.0*	0.909
Postoperative 3 month PH	27.8 ± 2.2	28.1 ± 4.0	0.887
Last follow-up AH	26.6 ± 2.3*	27.2 ± 2.5*	0.520
Last follow-up PH	26.9 ± 1.9*	28.0 ± 2.6	0.278
ADA change > 5°	8 (47.1%)	27 (33.8%)	0.405
Prosthesis–endplate depth ratio	0.91	0.90	0.866

*AHO* anterior heterotopic ossification, *ADA* arthroplasty disc angle, *ROM* range of motion, *FSU* functional spinal unit, *AH* anterior height, *PH* posterior height

* *P* < 0.05, compared with pre-operation or post-operation (1 week)

#### Radiographical changes at the replacement level

The arthroplasty disc angle in the AHO group and non-AHO group was maintained at 2.1° ± 5.2° and 3.4° ± 5.5° at the last follow-up, respectively, with no significant difference between the two groups (*P* = 0.651). Moreover, there were no significant differences in the mobility of CDA prosthesis between the two groups (*P* = 0.537). The paired-samples t test showed no statistically significant difference between pre-operation and the last follow-up in both 2 groups (*P* > 0.05).

#### The prosthesis–endplate depth ratio and changes in arthroplasty disc angle

We further detected the prosthesis–endplate depth ratio and found no significant association between prosthesis–endplate depth ratio and AHO occurrence (*P* = 0.405) (Table 4). In addition, we found that the rate of arthroplasty disc angle change > 5° in Group AHO (25.7%) was lower than that in Group non-AHO (33.9%). But statistical analysis showed no significant association (*P* = 0.866) (Table 4).

#### The incidence rate and changes in ABL and AHO

In the inter-observer reliability of ABL and AHO, the kappa values of the spine surgeons were determined as 0.91 and 0.87, respectively. As proposed by Landis et al. [22], with kappa values 0.81–1.00 considered as almost perfect agreement. The overall incidence rate of ABL was 44.3% (43/97). Severe ABL was rare and occurred in 8.2% (8/97) of all cases. Meanwhile, most of the ABL (36/43, 83.7%) occurred within the first 3 months (58.3% mild, 30.6% moderate, 11.1% severe). Among those, 25.0% (9/36) further developed a higher-degree ABL at 6 months, including 3 endplates collapse. Thereby, mild ABL occurred in 41.2%, moderate ABL in 42.9%, and severe ABL in 16.7% of CDA segments with peri-prosthesis bone loss at 6 months after surgery. On the other hand, the incidence rates of AHO at 3, 6, and 12 months post operation and during the final follow-up were 2.1, 7.2, 13.4 and 17.5%, respectively. The association of ABL and AHO is shown in Table 5. ABL was identified in 70.6% of AHO cases (33.3% mild, 41.7% moderate, 25.0% severe) and 38.8% of non-AHO cases (38.7% mild, 45.2% moderate, 16.1% severe), representing a statistically significant difference (*P* < 0.05). However, no significant difference was found between ABL degree and AHO (*P* = 0.094).

**Table 5:** Comparison of ABL degree between AHO and non-AHO group

Variables	AHO	Non-AHO	*P* Value
ABL (+ / -)	12 / 5	31 / 49	0.016
None	5	49	0.094
Mild	4	12	
Moderate	5	14	
Severe	3	5	

*AHO* anterior heterotopic ossification, *ABL* anterior bone loss

## Discussion

Theoretically, HS which combined ACDF at the spondylotic segment with CDA at the mobile segment should be intermediate between both ACDF and CDA in terms of intraoperative and postoperative results. Studies in recent years compared HS to ACDF or CDA and showed that HS could indeed acquire satisfactory outcomes [5–9]. In our study, with respect to clinical outcomes, a significant difference was found in postopearative JOA, NDI, and VAS scores compared with preopearative parameters in the AHO group and non-AHO group. Moreover, the present study demonstrated that AHO did not affect the clinical outcome. No significant differences in VAS, NDI, and JOA scores were observed between the two groups at the last follow-up. Furthermore, Kieser et al. [14] and Heo et al. [23] confirmed that ABL does not affect clinical outcomes. These results demonstrated that the relief of clinical symptoms depends on complete neurological decompression rather than the local curvature or segmental motion.

Regarding the cervical kinematic analysis, the postoperative CL of HS was significantly greater than preopeartion, which may related to the function of Zero-P implant. Wang et al. [24] found that the Zero-P implant could reinstate CL after surgery. According to previous biomechanical studies, theoretically, nonsurgical segment will compensate for the motion loss of the fusion segment to maintain ROM and decrease the abnormal hypermobility [5, 9, 25]. Similarly in this study, the postoperative ROM of the arthroplasty segment adjacent to fusion maintained as compensation for the fused segment. However, for the reason that the mobility of the cervical spine was reduced by a fusion segment, the C2–C7 ROM was statistically decreased (*P* < 0.05). Though study suggested that severe HO could restrict the mobility of replacement segments [15, 20] and the prevalence of ROM-limiting HO was 11.0% [16], this current study did not find that AHO limited the mobility of the replacement segment.

HO is defined as the formation of bone tissue outside the skeletal system. Certain surgeries or trauma such as total hip replacement and spinal cord injury can be complicated by HO. In 2005, Parkinson and Sekhon [10] firstly reported the occurrence of HO after CDA in a case study, and this phenomenon has since received increasing attention. According to a recent systematic review and meta-analysis of 5861 CDA prostheses, the overall pooled prevalence of HO was 32.5% (95% CI 26.7 to 38.4%) [16]. However, different prostheses have their distinct biomechanical characteristics, design, and implantation techniques which have been postulated to contribute to the formation of HO [26, 27]. And the HO incidence rate of Prestige-LP has been reported to range from 31.3 to 41.9% [13, 19, 20], compared with 66.0% (64/97) in the current study. The behavior of the arthroplasty level adjacent to fusion in HS could be more severely affected than that in stand-alone CDA [5, 28]. Our previous study also revealed that the HO incidence rate in 2-level HS groups was higher than for that of the 1-level CDA [13]. We thus infer that the overall HO occurrence in HS differs from single-level CDA. Furthermore, AHO was identified in 17.5% (17/97) of all CDAs in our study. Similarly, Tian et al. [15] conducted an analysis of paravertebral ossification in 82 CDAs including multilevel CDAs using CT scan and showed that AHO was only identified in 19.1% of all prostheses.

Regarding the factors affecting HO, in addition to the fusion segments and prosthesis types, older age, male sex, operative level, and genetic predisposition have also been reported in different studies [16, 19, 27]. Furthermore, a retrospective study has shown that the incidence and severity of HO increased in a longer follow-up time [26]. Nonetheless, as shown in our results, the AHO occurrence exhibited no significant correlation with age, gender, follow-up time, or involved level. Meanwhile, changes to the local alignment and balance during prosthesis implantation might be involved in the occurrence and development of HO [19, 21, 29]. Hu et al. [19] suggested that the probability that ROM-limiting HO occurred in the group with a more than 5° disc space angle lordosis increase was significant greater. And Tu et al. [29] concluded that inadequate endplate coverage and shell kyphosis have adverse effects on the formation of HO. There were more immobile (range of motion < 3°) artificial discs in the suboptimal carpentry group than the optimal carpentry group. However, only 8.9% of all ROM-limiting HO happened as AHO [15]. In our study, we calculated the ratio of the depth of prosthesis to endplate and change in disc space angle for comparison between the two groups. No obvious direct relationship between the occurrence of AHO and those two parameters was found. Therefore, the mechanism of AHO formation may be different from the lateral and posterior HO.

In the present study, the ABL occurrence rate was significantly higher in the HO group than in the non-HO group (70.6% versus 38.8%). This indicated that AHO was more prone to occur in segments where ABL appeared. Kim et al. [17] observed anterior bony ingrowth of the endplates into the adjacent device surface occurs with stability after ABL in the subacute recovery period. It explained why ABL was no longer progressive, as there was no more stress against this cortex with HO of the device into the intervertebral space. According to Wolf ‘s law, the trabecular bone adapts to mechanical stimuli based on observations of the self-optimizing bone property. In addition, Marco et al. [30] showed that after the initial inflammatory phase which follows implant insertion, the subsequent bone regeneration process is strongly influenced by the implant. The forces needed to insert the implant can cause a fairly high amount of microdamage away from the implant surface [30] which, in turn, triggers a substantial but short-term increase in peri-prosthesis bone resorption followed by formation [31]. Postoperative CT images obtained over 2 years after CDA also depicted that there was not bone loss of the vertebrae but rather bone remodeling [23]. Herein, most of the ABL (36/43, 83.7%) occurred within the first 3 months. On the contrary, more patients (10/17, 58.8%) occurred with AHO after the first 6 months. The incidence correlation as well as temporal relationship indicated that AHO may be as a result of the bone healing process, which eliminates the instability caused by ABL. Thus, we postulated ABL and AHO may consist of two successive progress of bone reconstruction after HS.

However, excessive bone loss may lead to prosthesis subsidence [21] (Fig. 1), and severe HO could obviously affect the motion-maintaining function of CDA (Fig. 3), contrary to its design philosophy. Therefore, the balance of the bone reconstruction process are essential to avoid complications after CDA and HS. The study also suffers from some other limitations. First, the retrospective nature of our study may be associated with bias, especially in radiographical measurements. Second, different prostheses with distinct biomechanical characteristics may be diverse in bone reconstruction. Third, comparisons of characteristics among the different ABL-degree groups need further attention. A specific study design with multivariate analysis of large-scale and longer follow-up would be important.

## Conclusions

The study confirmed that ABL was common in HS, and it was related to AHO. However, there was no significant correlation between different ABL degrees and AHO. The formation of AHO and ABL could be integral parts of anterior bone remodeling after cervical spine surgery.

## Data Availability

Summarized data have been presented in this manuscript. The raw data for this study are located and protected at West China Hospital of Sichuan University. Sharing of the raw data is not suggested, because a secondary analysis is planned.

## Abbreviations

CDA: Cervical disc arthroplasty
ACDF: Anterior cervical discectomy and fusion
HS: Hybrid surgery
CDDD: Cervical degenerative disc disease
HO: Heterotopic ossification
ABL: Anterior bone loss
AHO: Anterior heterotopic ossification
CT: Computed tomography
MRI: Magnetic Resonance Imaging
JOA: Japanese Orthopedics Association
NDI: Neck Disability Index
VAS: Visual Analogue Scale
ROM: Range of motion
CL: Cervical lordosis
FSU: Functional spinal unit
SD: Standard deviation
IQR: Interquartile range

## References

[1] Theodore N (2020). Degenerative cervical Spondylosis. N Engl J Med.

[2] Phillips FM, Geisler FH, Gilder KM, Reah C, Howell KM, McAfee PC (2015). Long-term outcomes of the US FDA IDE prospective, randomized controlled clinical trial comparing PCM cervical disc Arthroplasty with anterior cervical discectomy and fusion. Spine (Phila Pa 1976).

[3] Gornet M, Burkus J, Shaffrey M, Schranck F, Copay A (2019). Cervical disc arthroplasty: 10-year outcomes of the prestige LP cervical disc at a single level. J Neurosurg Spine.

[4] Radcliff K, Davis RJ, Hisey MS, Nunley PD, Hoffman GA, Jackson RJ, Bae HW, Albert T, Coric D (2017). Long-term evaluation of cervical disc Arthroplasty with the Mobi-C© cervical disc: a randomized, prospective, multicenter clinical trial with seven-year follow-up. Int J Spine Surg.

[5] Jia Z, Mo Z, Ding F, He Q, Fan Y, Ruan D (2014). Hybrid surgery for multilevel cervical degenerative disc diseases: a systematic review of biomechanical and clinical evidence. Eur Spine J.

[6] Ren X, Chu T, Jiang T, Wang W, Wang J, Li C, Zhang Z (2016). Cervical disk replacement combined with cage fusion for the treatment of multilevel cervical disk herniation. Clin Spine Surg.

[7] Shin DA, Yi S, Yoon DH, Kim KN, Shin HC (2009). Artificial disc replacement combined with fusion versus two-level fusion in cervical two-level disc disease. Spine (Phila Pa 1976).

[8] Wang K-F, Duan S, Zhu Z-Q, Liu H-Y, Liu C-J, Xu S (2018). Clinical and radiologic features of 3 reconstructive procedures for the surgical Management of Patients with Bilevel cervical degenerative disc disease at a minimum follow-up period of 5 years: a comparative study. World Neurosurg.

[9] Grasso G (2015). Clinical and radiological features of hybrid surgery in multilevel cervical degenerative disc disease. Eur Spine J.

[10] Parkinson JF, Sekhon LH (2005). Cervical arthroplasty complicated by delayed spontaneous fusion. J Neurosurg Spine.

[11] Tumialán LM, Gluf WM (2011). Progressive vertebral body Osteolysis after cervical disc Arthroplasty. Spine (Phila Pa 1976).

[12] Park J-B, Chang H, Yeom JS, Suk K-S, Lee D-H, Lee JC (2016). Revision surgeries following artificial disc replacement of cervical spine. Acta Orthop Traumatol Turc.

[13] Hu L, Wu T, Liu H, Wang B, Zhang J, Meng Y, Ding C, Gao X, Hong Y (2019). Influence of fusion on the behavior of adjacent disc Arthroplasty in contiguous 2-level hybrid surgery in vivo. World Neurosurg.

[14] Kieser D, Cawley D, Fujishiro T, Mazas S, Boissiere L, Obeid I, Pointillart V, Vital J-M, Gille O (2018). Risk factors for anterior bone loss in cervical disc arthroplasty. J Neurosurg Spine.

[15] Tian W, Fan M-X, Liu Y-J, Han X, Yan K, Wang H, Lyu Y-W (2016). An analysis of paravertebral ossification in cervical artificial disc replacement: a novel classification based on computed tomography. Orthop Surg.

[16] Hui N, Phan K, Kerferd J, Lee M, Mobbs RJ (2019). Prevalence of and risk factors for heterotopic ossification after cervical Total disc replacement: a systematic review and meta-analysis. Glob Spine J.

[17] Kim SH, Chung YS, Ropper AE, Min KH, Ahn TK, Won KS, Shin DA, Han IB (2015). Bone loss of the superior adjacent vertebral body immediately posterior to the anterior flange of Bryan cervical disc. Eur Spine J.

[18] Sevastikoglou JA, Bergquist E (1969). Evaluation of the reliability of radiological methods for registration of scoliosis. Acta Orthop Scand.

[19] Hu L, Zhang J, Liu H, Meng Y, Yang Y, Li G, Ding C, Wang B (2019). Heterotopic ossification is related to change in disc space angle after prestige-LP cervical disc arthroplasty. Eur Spine J.

[20] Zeng J, Liu H, Chen H, Rong X, Meng Y, Yang Y, Deng Y, Ding C (2019). Effect of prosthesis width and depth on heterotopic ossification after cervical disc Arthroplasty. Spine (Phila Pa 1976).

[21] Kieser DC, Cawley DT, Fujishiro T, Tavolaro C, Mazas S, Boissiere L, Obeid I, Pointillart V, Vital JM, Gille O (2019). Anterior bone loss in cervical disc Arthroplasty. Asian Spine J.

[22] Landis JR, Koch GG (1977). The measurement of observer agreement for categorical data. Biometrics.

[23] Heo DH, Lee DC, Oh JY, Park CK (2017). Bone loss of vertebral bodies at the operative segment after cervical arthroplasty: a potential complication?. Neurosurg Focus.

[24] Wang C, Zhang Y, Yuan W (2016). Early clinical outcomes and radiographic features after treatment of cervical degenerative disk disease with the new zero-profile implant: a 1-year follow-up retrospective study. Clin Spine Surg.

[25] Schwab JS, DiAngelo DJ, Foley KT (2006). Motion compensation associated with single-level cervical fusion: where does the lost motion go?. Spine (Phila Pa 1976).

[26] Yi S, Kim KN, Yang MS, Yang JW, Kim H, Ha Y, Yoon DH, Shin HC (2010). Difference in occurrence of heterotopic ossification according to prosthesis type in the cervical artificial disc replacement. Spine (Phila Pa 1976).

[27] Yi S, Oh J, Choi G, Kim TY, Shin HC, Kim KN, Kim KS, Yoon DH (2014). The fate of heterotopic ossification associated with cervical artificial disc replacement. Spine (Phila Pa 1976).

[28] Eck JC, Humphreys SC, Lim TH, Jeong ST, Kim JG, Hodges SD, An HS (2002). Biomechanical study on the effect of cervical spine fusion on adjacent-level intradiscal pressure and segmental motion. Spine (Phila Pa 1976).

[29] Tu T-H, Wu J-C, Huang W-C, Guo W-Y, Wu C-L, Shih Y-H, Cheng H (2011). Heterotopic ossification after cervical total disc replacement: determination by CT and effects on clinical outcomes. J Neurosurg Spine.

[30] Marco F, Milena F, Gianluca G, Vittoria O (2005). Peri-implant osteogenesis in health and osteoporosis. Micron.

[31] Wang L, Ye T, Deng L, Shao J, Qi J, Zhou Q, Wei L, Qiu S (2014). Repair of microdamage in osteonal cortical bone adjacent to bone screw. PLoS One.

